# Adjustment of *p*-value expression to ontology using machine learning for genetic prediction, prioritization, interaction, and its validation in glomerular disease

**DOI:** 10.3389/fgene.2023.1215232

**Published:** 2023-10-12

**Authors:** Boutaina Ettetuani, Rajaa Chahboune, Ahmed Moussa

**Affiliations:** ^1^ Systems and Data Engineering Team, National School of Applied Sciences, Abdelmalek Essaadi University, Tétouan, Morocco; ^2^ Life and Health Sciences Team, Faculty of Medicine and Pharmacy, Abdelmalek Essaadi University, Tétouan, Morocco

**Keywords:** glomerular diseases, gene expression, gene ontology, machine learning, ETL

## Abstract

The results of gene expression analysis based on *p*-value can be extracted and sorted by their absolute statistical significance and then applied to multiple similarity scores of their gene ontology (GO) terms to promote the combination and adjustment of these scores as essential predictive tasks for understanding biological/clinical pathways. The latter allows the possibility to assess whether certain aspects of gene function may be associated with other varieties of genes, to evaluate regulation, and to link them into networks that prioritize candidate genes for classification by applying machine learning techniques. We then detect significant genetic interactions based on our algorithm to validate the results. Finally, based on specifically selected tissues according to their normalized gene expression and frequencies of occurrence from their different biological and clinical inputs, a reported classification of genes under the subject category has validated the abstract (glomerular diseases) as a case study.

## 1 Introduction

C3 glomerulopathies (C3G) are a group of related conditions that cause kidney dysfunction (Riedl et al., 2017), characterized by the presence of glomerular deposits composed of C3 (Cook and Pickering, 2015). Many conditions in glomerular diseases (GD) have a variety of genetic/environmental causes (Coelho et al., 2019). C3G is associated with changes (mutations) in many genes. Most of these genes provide instructions for making proteins that help regulate a part of the body’s immune response known as the complement system (Iatropoulos et al., 2016). This system works together as a group of proteins to destroy foreign invaders/triggers/inflammation. The complement system must be regulated, targeting all unwanted materials without damaging the body’s healthy cells. A specific mutation in the complement system-related genes, like C3, ADAM19, ADAMTS13, C3AR1, C8A, CD46, CFB, CFD, CFI, CFHR (1-5), in addition to other complement system-related genes (Tsai et al., 2000; Xiao et al., 2014), risk haplotypes of CFH and CD46 have been identified that modify disease penetration and severity (Legendre et al., 2013; de Cordoba et al., 2014). In most cases, the cause of the C3G is unknown.

Many kinds of research are still devoted to discovering genes involved in specific phenotypes and diseases. Multiple gene selection techniques are defined in the literature. Whichever confidence in using a single criterion for selecting genes is not always adopted which specific used one should be diffident.

**FIGURE 1:**
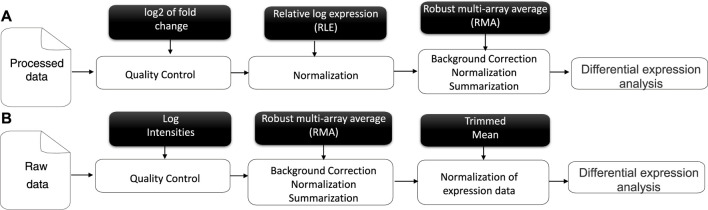
Literature review of Affymetrix microarray data pre-processing for processed **(A)** and raw data **(B)**.

This question inspired us to consider the ranking of all criteria in the evaluation of the gene and propose a new selection of genes for transcriptomic data, focusing on the gene expression adjustment to the similarity score. Thus, the genes for each criterion would be systematically computed and validated by our algorithm. Our solution can be considered the most informative and stable method for gene prediction/selection and classification steps. In the meta-analysis process, the input of gene expression results consisted of normalized gene expression measurements. From this, a linear model fit for all genes of our transcriptomics data can be computed as an appropriate contrast function to test hypotheses of interest and to find genes with significant differential expression (DE) between different conditions (Klaus and Reisenauer, 2016) from understudied raw data (as a set of binary files in CEL format), accessible via the public repository of microarray data, the NCBI Gene Expression Omnibus (GEO) (Clough and Barrett, 2016). The raw data were chosen to be used as extracted from the source rather than processed data, although their analysis is very similar, as mentioned in (Figure 1) representing a literature review. The first step in pre-processing is data quality control. The latter is an essential step in any analytical process and a relative concept that depends on the nature of the biological sample, experimental settings, and other factors. Hence, poor-quality data can directly lead to the absence of some positive results. Moreover, we evaluated the measure of precision to reduce deficiencies over time and under varying operating conditions (Bolstad et al., 2003; Kauffmann et al., 2009; Carvalho and Irizarry, 2010). Different normalization methods have been developed in the context of gene expression analysis. A specific normalization method in microarray data analysis is crucial to ensuring accurate and reliable results. RMA (Robust Multi-array Average) was chosen over other methods such as MASS, GCRMA, PLIER, PUMA, etc.

Microarray data and RNA-Seq data are generated through different technologies and have distinct characteristics. Microarrays measure the relative abundance of pre-selected probes for known genes, while RNA-Seq directly sequences and quantifies the transcriptome, including known and novel transcripts. Initially developed for microarray data, the RMA algorithm can be applied to RNA-Seq data with different disease modalities and normalization methods, offering new insights into gene expression analysis for different biological contexts. While some concepts and principles from microarray data analysis may be relevant to RNA-Seq analysis, it is crucial to use appropriate RNA-Seq-specific methods to accurately handle the data and obtain reliable results.

The RMA algorithm was performed on our data to background-correct, normalize, and summarize the process (Okoniewski and Miller, 2006; McCall and Irizarry, 2011), offering several advantages, such as reducing the impact of extreme values. The choice of RMA over other methods depends on the specific characteristics of the dataset and the specific research questions of the analysis. RMA is often favored due to its robustness and simplicity compared to other methods like GCRMA, PLIER, or PUMA that might be more suitable.

The organization of this article is presented in two main sections, according to the following principles. The first one (methods) includes gene signature identification in connection with the search for a therapeutic target involved in the detection of differentially expressed (DE) genes, followed by a subsequent step leading to the construction of the workflow and its structure to prioritize and interact with a gene on each cluster based on Expression-Similarity-Frequency of occurrence. A second section (results and discussion) covers the interpretation of the biological/clinical results as a form of evaluation and validation of our hypothesis.

## 2 Materials and methods

**FIGURE 2:**
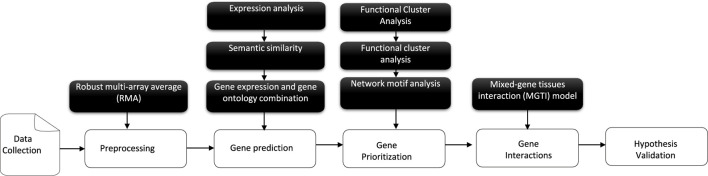
An overview of the schematic workflow of the developed approach, consisting of five components: first, transcriptomics data collection and pre-processing steps; then, gene prediction and prioritization. Finally, a human genome-scale genetic interaction model is followed by hypothesis evaluation and validation.

The main focus of this follow-up study (Figure 2) is to propose and validate a novel matrix-expression-similarity-frequency consisting of a new scoring scheme based on a given combined/adjusted linear DE measurement selection in diverse experimental conditions for individual samples. Machine learning tasks then combine a mathematical algorithm with our prediction results analysis (Tarca et al., 2007; Xu and Jackson, 2019; Sabir et al., 2021). The gene cluster lists suggested using unsupervised learning based on their normalized matrix-expression-similarity-frequency-based scores of occurrences first to find out the structure as groups of a similar category. In this case, the data contains only inputs and no desired output labels. Then, classification is the second step in which the algorithm keeps in check both the inputs and the desired outputs (a limited set of outputs) to construct co-regulation and link them into networks that prioritize candidate genes as a logistic regression tool. In addition, significant genetic interactions for a specific tissue type, genetic background, experimental stimulus, or clinical variable are detected and validated in the results.

### 2.1 A computational algorithm for gene correction

The statistical methods used to detect DE genes were calculated as moderated t-statistics for the microarray data based on a linear model fit by fixing three different *p*-values (p1 = 0.01, p2 = 0.001, p3 = 0.0001), which are estimated as the prior probability that a gene is DE (Jeffery et al., 2006; Sartor et al., 2006). It should be noted that when we fixed a different *p*-value distribution to our dataset, we demonstrated that the expression of these candidate genes between these *p*-values is expected to change the methods employed in GD diagnosis and prognosis. The semantic similarity computation was assumed based on the Wang method (Wang et al., 2007), and using the GOSemSim (Yu et al., 2010) package between gene products based on the information content (IC) and a directed acyclic graph (DAG) (Mazandu and Mulder, 2013). The IC-based measures depend on the frequencies of two GO terms involved in their closest common ancestor term in a specific corpus of GO annotations. The GO terms were classified into three different aspects: molecular function (MF), biological process (BP), and cellular component (CC). The molecular function is a process extended by two actions described as biochemical binding activities, referring to a protein that functions as a receptor. The second aspect is that the biological process represents an organism’s specific and significant objective (Gaudet et al., 2017; Thomas, 2017). Finally, the cellular component, in terms of cellular structures and location, provides information about where a molecular process may occur. The best-match average (BMA) method calculates the average of all maximum similarities on each row and column. Furthermore, a new score for the gene similarity measures was calculated for each pair of genes as demonstrated here (Eq. 1), through which the matrix evaluates whether the mean and normally distributed score within each independent pair of genes of samples evaluates an important significance or not by introducing the matrix-similarity-based.

$$M_{\mathrm{simsc}}\left(g_{1},g_{2}\right)=\overset{\mathrm{Lengthx}}{\underset{n=1}{\sum }}\overset{\mathrm{Lengthx}}{\underset{n=n+1}{\sum }}\left(\sqrt{\frac{1}{2}*\left(\frac{\delta_{1}^{2}}{\delta_{2}^{2}}+\frac{\delta_{2}^{2}}{\delta_{1}^{2}}\right)}\right)*{\left(\frac{\mu_{1}+\mu_{2}}{2}\right)}^{2}$$ (1)

• *M*_*simsc*_ represents the similarity of the gene measurement matrix.
• Only paired groups of genes can perform the paired test.
• Based on the first two DE genes, *∑*_*n*=1_ *∑*_*n*=*n*+1_ up to the full set of genes as mentioned in *Lengthx* were selected for *M*_*simsc*_.
• The sample means are denoted as *μ*_1_ and *μ*_2_ for each similarity score.
• Each score is sampled independently and randomly.
• The sample standard deviations *δ*_1_ and *δ*_2_ are normally distributed within each of the two rows.

Gene prediction model (based on their gene expression and gene-GO similarity) represented as matrix-expression-similarity-frequency-based consisting of a new adjusted scoring scheme of the score and frequencies (as results) for a given linear DE measurement selection results mixed with their scores and frequencies of occurrence of matrix-similarity-based, which yield the final association score. The genes with the highest scores were first selected and improved to serve as inputs for the machine learning steps.

$$M_{\mathrm{CombSc}}=n_{\mathrm{expr}}*M_{\mathrm{expr}}+n_{\mathrm{sel}}*M_{\mathrm{simsc}}$$ (2)

• A number of expressed genes *n*_*expr*_ provided with fixed *p*-values *M*_*expr*_ were combined into the expression matrix.
• A number of correlated genes *n*_*sel*_ were combined into the semantic similarity matrix *M*_*simsc*_.

### 2.2 Prioritization of candidate genes based on machine learning

Machine learning methods (Ganggayah et al., 2019; Zhang, 2020; Ke et al., 2021) were first performed as clustering algorithms (Chou et al., 2007; Maulik et al., 2009), building a mathematical model from the normalized final scores of our matrix-expression-similarity-frequency-based (Eq. 2) to segment them into k clusters to understand biological processes in addition to molecular functions. Each cluster represents a group of similar observations performed using the Ward method and Euclidean distance for a given value of k as a possible solution (i.e., high intra-class similarity). Although objects from different clusters are as dissimilar as possible (i.e., low inter-class similarity), to improve the initial partition obtained from hierarchical clustering. The algorithm can stop when the assignment of genes to clusters no longer changes or when the specified maximum number of iterations has been reached (Gry et al., 2009; de Winter et al., 2016). Justifying the choice of distance metric and comparing the results with several known distances are essential steps that can significantly impact the results and ensure the robustness and reliability of the machine learning model, including candidate gene classification. The appropriateness of the Euclidean distance depends on the nature of the data and the problem at hand for candidate gene classification:

The classical methods for distance measures are Euclidean, Manhattan, and correlation-based distances, used for gene expression, such as Pearson correlation distance and Spearman correlation distance. The correlation-based distance considers two similar objects if their features are highly correlated. The convergence between the Manhattan distance and Euclidean distance for gene expression depends on some specific characteristics and distributions of gene expression data. The lasts are widely used to measure the similarity or dissimilarity between samples based on their gene expression profiles. In summary, the choice between Manhattan and Euclidean distances for gene expression data should consider the vector space (dimensionality) of the data and the number of genes being analyzed. When the number of genes is relatively small due to the pre-processing and DE analysis, both Manhattan and Euclidean distances may behave similarly, especially if the genes are highly correlated or there is little variability in the data. Additionally, it was essential to experiment with different metrics and compare their performance using appropriate evaluation techniques such as cross-validation to select the best distance metric, which is why we tried all the discussed methods, and the results are accessible in our two previous published papers (Ettetuani et al., 2019; Ettetuani et al., 2020). Finally, the Euclidean distance assumes that all features have the same scale and are equally important. Euclidean distance treats each feature independently, without considering correlations between them.

Further, each gene cluster list was exposed to the (hgu133a,hgu133plus2) database of *Homo sapiens* as a direct mapping of a gene symbol to a vector containing the corresponding Entrez gene identifier (Smedley et al., 2009; Yu et al., 2012), then implemented in a hypergeometric model to assess whether the number of selected genes is linked/associated with the pathogenesis of the diseases (Yu, 2012; Fabregat et al., 2018). All enriched terms were associated with their enrichment scores (*p*-values) as a first step of supervised learning, allowing the possibility to cross from high-level concepts to detailed pathway diagrams showing bio-molecular events using the groupGO(), and enrichGO() functions (Sidiropoulos et al., 2017; Wu et al., 2021). Genetic variation was also studied through our first step of supervised learning, which is the genome-wide association study (GWAS); based on the GWAS catalog used to tag variation across the genome and enable investigations to identify causal variants (Johnson et al., 2010; Scharf et al., 2013; Butler et al., 2017; Garfield, 2020) and variant-trait associations mapped to their chromosomal positions in the human genome. Many computational approaches have been developed to support the identification of the most promising candidates (Zolotareva and Kleine, 2019). oPOSSUM (Ho Sui et al., 2007) web applications containing a great variety of the conserved non-coding regions of the promoters/enhancers were used to select the interaction between our candidate’s genes, whose interactions between genes and transcription factors (TFs) were a major to understand gene regulation and the origin of complex protein components (Suravajhala and Benso, 2017).

To facilitate the prioritization of causative genes as the second step of supervised learning and based on algorithmic tools, we illustrate a new gene prioritization model for candidate genes based on the logistic regression method (Lee et al., 2018; Zhang et al., 2018; Christodoulou et al., 2019; Nusinovici et al., 2020). Gene prioritization schemes boost the power to identify the most promising phenotype-associated among those clusters under normal conditions in different tissues [where each gene can have a normalized expression score in the tissue expression database (Palasca et al., 2018)].

$$Score_{\mathrm{prioritization}}\left(\left.Gene_{i}\right|Tissue_{t}\right)=\mu_{0}+\mu_{1}X_{1}+\mu_{2}X_{2}+\mu_{3}X_{3}+\cdots +\mu_{n}X_{n}$$ (3)

• *μ*_0_ is the tissue-specific means of expression for a given gene with fixed tissues.
• *μ*_1_, *μ*_2_, *μ*_3_, . .*μ*_*n*_ are the means of (frequencies of occurrence) for target genes for each process.
• X1, X2, X3, , Xn represent the normalized expression values of biological processes, GWAS, TFs, etc.
• The parameters in logistic regression cannot simply be replaced by average values, especially when the output is a probability-related value. The parameters in logistic regression represent the relationship between the input variables (in this case, gene expression means) and the probability of normalized expression values based on biological processes.
• Replacing the parameters with average values would be highly unusual and would be approved later by the methodology results.

Following the hypothesis that genes underlying similar tissues will share functional and phenotypic characteristics, we incorporated logistic regression for any training genes that need to be prioritized (Eq. 3). When applying logistic regression, it is generally recommended to split the available data into three separate sets: a training set to estimate the model parameters, a validation set to tune the model hyperparameters and assess its performance, and a test set. The formulated model arranged the candidate genes (i) in the order of their tissues (t) first to be sure they were associated with the pathology. The algorithm took two inputs: a collection of evidence sources defining a phenotype/trait of interest, and enhancer/promoter interaction information, extracted for a given gene, linked to each other with a normalized score reflecting the “likelihood” for each gene to be responsible for the phenotype. Then a second factor; frequencies of occurrence of each entire represents a mean of the prioritization factor. The output of the algorithm resulted in a list of candidate genes arranged according to the calculated scores for each tissue.

**FIGURE 3:**
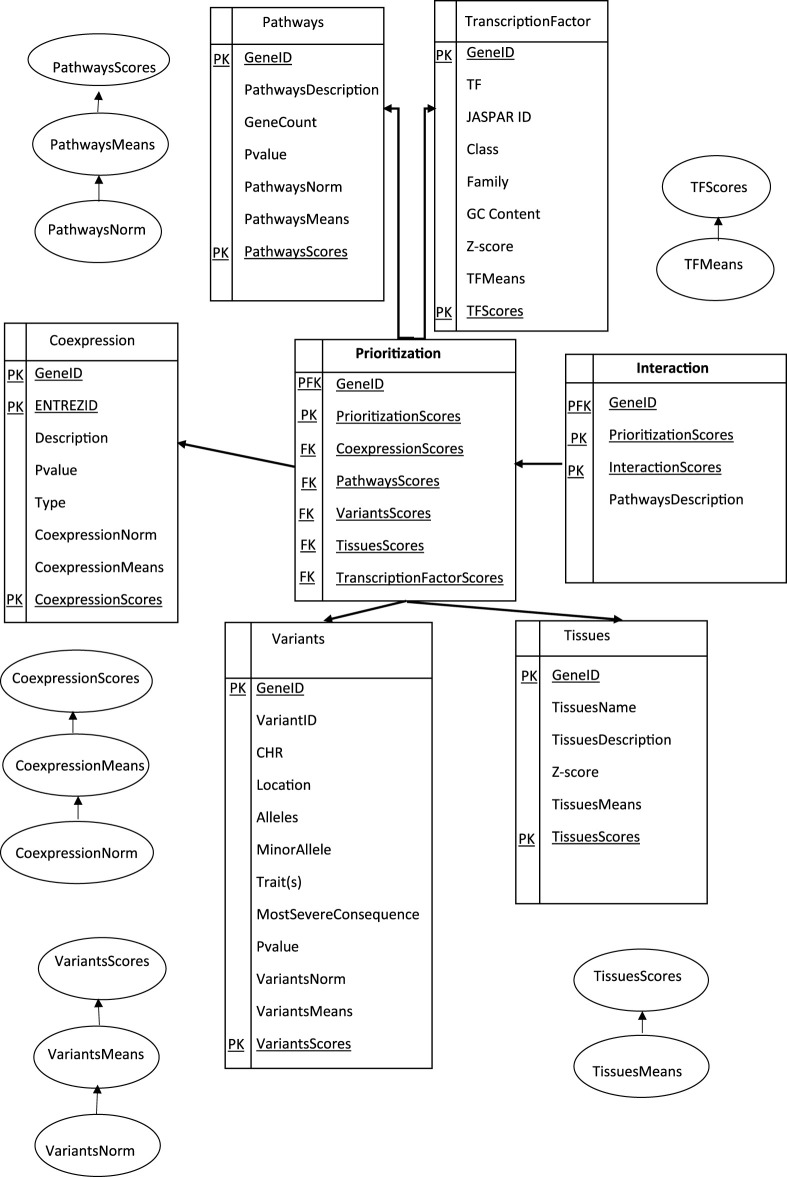
Data Warehouse Star Schema represented in SQL: A fact table representing the prioritization and interaction model for different dimension tables. Data were collected based on Eq. 3, stored in SQL databases and structured in tables with predefined schemas.

Overall, the process of extracting, transforming, and loading ETL data from homogeneous or heterogeneous sources was established to access our data (Biswas et al., 2020; Biswas et al., 2018). In short, it was an essential component in cleansing, customizing, reformatting, integrating, and inserting the prerequired data (Greiff et al., 2015; Tecnico, 2015). In this paper, we tried to navigate through our adjusted genes to conceptualize the ETL processes, as shown in (Figure 3), first modeling a prioritization tool, then modeling genetic interaction constructs into proper storage (format/structure) for querying and analysis.

The Primary Key (PK) uniquely identified each row in an interactive table. In the star schema, the dimension tables were typically designed with surrogate keys that were independent of the source data. These surrogate keys were used to establish relationships between the schema dimensions and the fact table(s). A proper star schema design often includes a central fact table that contains the Primary Foreign Key (PFK) of the dimension tables, along with the numerical values (facts) associated with those dimensions as a combination of a primary key and a foreign key in a database. It was used to establish a relationship between different tables, allowing and creating a link between a table to reference another table’s primary key. The dimension tables have descriptive attributes that provide additional information about the dimensions. A collection of candidate geneIDs arranged according to a specific tissue (kidney and urine and Immune system and blood and Embryonic dev) with the highest information of the gene related to specific biological pathway terms, the gene-variant-trait associations among the GWAS catalog, and the genes and transcription factors (TFs) interaction are the most efficient approaches to representing genomic data. A Foreign Key (FK) column in the prioritization table refers to the primary key in another table to create our study a connection between each calculated score-related data, allowing for data retrieval and enforcing referential integrity.

One way to understand these terms and enforce data integrity involves defining the structure and relationships based on primary and foreign keys (PK and FK). Primary keys uniquely identify each record in a table, while foreign keys establish relationships between tables to design, build, and manage efficient and reliable databases, preventing invalid or inconsistent data from being inserted or updated.

### 2.3 Gene interaction

**FIGURE 4:**
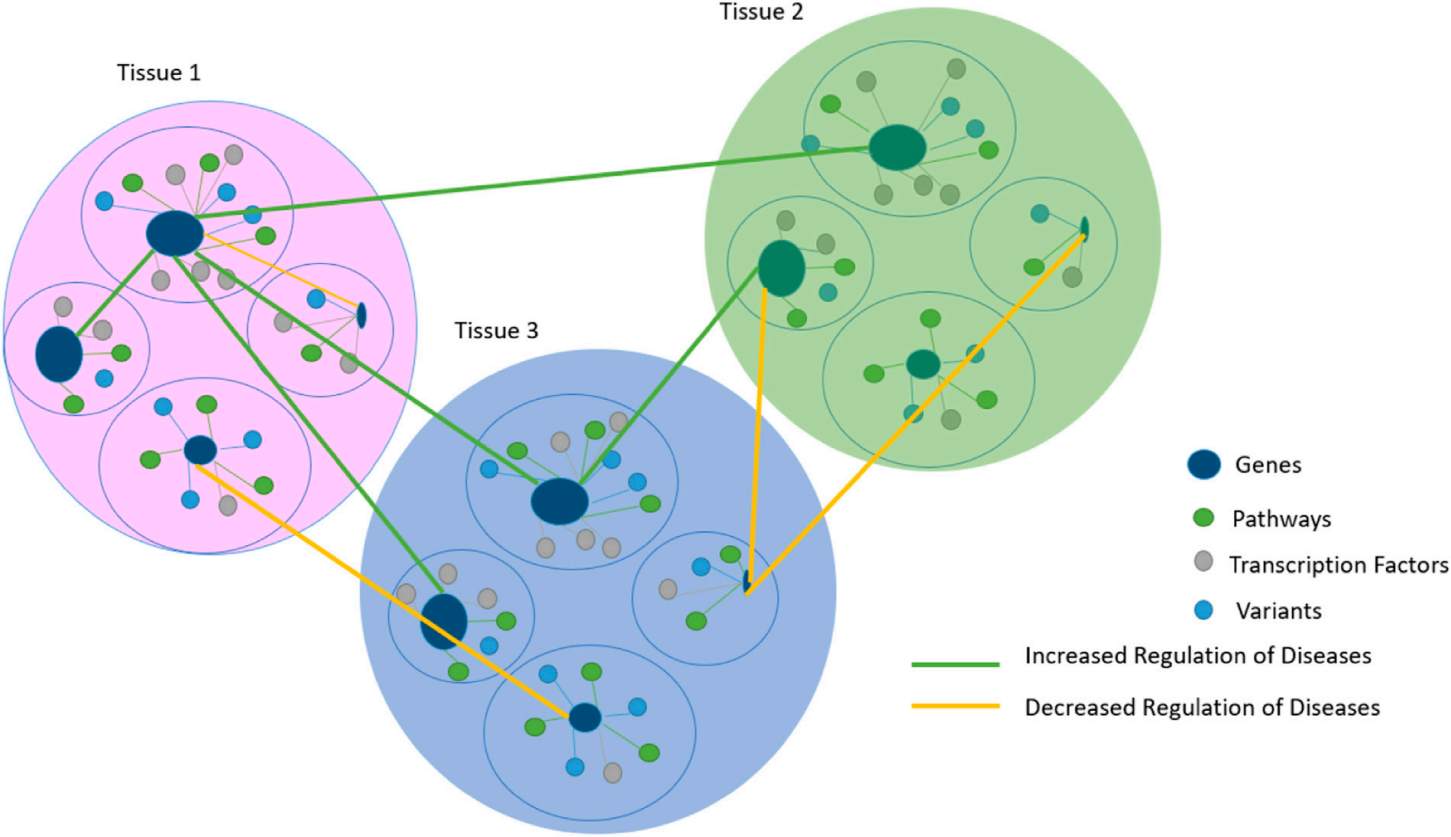
Gene prioritization results are based on tissues that are related to each other by regulating disease links. The prioritization scheme in Figure 4 is used to facilitate the comprehension of different Supplementary Figures and serves as a starting point for the sequence in Figure 3.

Genetic interactions of omics data refer to a combination of pairs of genes in different tissues of fixed clusters, as shown in (Figure 4), whose contribution to a phenotype between specific variants in complex traits and tissues remains a challenge (Gomez-Cabrero et al., 2014; Vasaikar et al., 2018; Subramanian et al., 2020).

A novel algorithm-based model called Mixed-Gene Tissue Interaction (MGTI) was developed based on previous data. Gene prioritization scores were first calculated, and then significantly interacted mixed genes between the selected tissues were identified using (Eq. 4), reflecting the strengths of regulatory interactions to understand the etiology of our glomerular disease as a use case.

$$Score_{\mathrm{inter}}\left(g_{1},g_{2}\right)=\frac{\log_{10}\left(S_{p}\left(g_{1}\right)\right)+\log_{10}\left(S_{p}\left(g_{2}\right)\right)}{2+\log_{10}\left(\max\left(S_{p}\left(g_{1},g_{2}\right)\right)\right)}$$ (4)

• *Score*_*inter*_(*g*_1_, *g*_2_) represents an interaction score for each pair of genes.
• Each gene is represented by the prioritization score *S*_*p*_, which is based on multiple calculated scores as mentioned in Eq. 3.
• max(*S*_*p*_(*g*_1_, *g*_2_)) represents the maximum gene prioritization score between the two selected genes.
• The interaction scores are between (0, 1).
• When the interaction scores are *≅* (0.31, 1), the MGTI model reflects an increased regulation of disease.
• When the interaction scores are *≅* (0.1, 0.3), the MGTI model reflects a reduced regulation of disease, where we should interact more with other possible genes.
• There is no gene score between *≅* (0, 0.1), because each gene is selected following a selection score and then validated to have n minimum pathway information in the prioritization section.

Moreover, as the number of interacting genes increases, traditional statistical methods are limited in their ability to identify interacting genes in high-dimensional data (Yang et al., 2011; Gordon et al., 2020; Sun et al., 2020).

## 3 Results and discussion

The detection of significant genetic interactions was focused on large-scale studies based on a selection of gene expression mixed with multiple similarity scores of their gene ontology (GO) terms. As well as their sources of biological became widely adopted. Our algorithm adjusted them to evaluate the regulation and link them into networks that prioritize candidate genes as classification by applying machine learning techniques related to glomerular diseases (GD).

The spectrum of glomerular diseases is defined by the abnormal control of complement cascade activation, whose actions are considered part of the innate immune system, procuring an immune complex deposition of fragments of C3 in glomeruli (Pickering et al., 2013). GD often results in kidney damage, the cause of which is unknown.

The first thing to do to measure the level of transcriptional genes was to validate the information stored in the raw data measurements (Dalman et al., 2012; Kharchenko et al., 2014), which were analyzed as described in our paper (Ettetuani et al., 2019), published by the ACM organization, Proceedings of the New Challenges in Data Sciences: Acts of the Second Conference of the Moroccan Classification Society, as a validation process of the information stored in expression sets. In this meta-analysis approach, each experiment was first analyzed separately, and all the results were then combined based on their primary statistics (*p*-values) (Walsh et al., 2015). Here, we fit the linear model for all genes and defined appropriate contrast functions to test hypotheses of interest to find genes with significant DE within each condition.

### 3.1 Experimental data

Based on the PubMed database, for Data retrieval, summarizing and comparing topics according to their frequencies of occurrence (Rani and Ramachandran, 2015; Gusenbauer and Haddaway, 2020), to broaden or/and narrow a search Kovalchik (2015), and to exclude unwanted search terms/concepts from a specific speech as “glomerulopathies” “diabetic kidney disease” “tumor Nephrectomy” “diabetic nephropathy” “focal segmental glomerulosclerosis” “rapidly progressive glomerulonephritis” “minimal change disease” and “membranous glomerulonephritis.”

In addition, five datasets (Table 1) were extracted consisting of human kidney biopsies of patients are used in our analysis, providing a comparison of the glomerular transcriptome for multiple profiles as the adult-onset steroid-sensitive focal segmental glomerulosclerosis and minimal change disease (Tong et al., 2015), transcriptomic and proteomic profiling reveals insights of mesangial cell function in patients with IgA nephropathy (Liu et al., 2017), glomerular transcriptome from subjects in the NEPTUNE cohort (Mitrofanova et al., 2018), and shared molecular targets in the glomerular transcriptome from patients with nephrotic syndrome and ANCA-associated vasculitis, and glomerular transcriptome from European renal cDNA bank subjects and living donors (Grayson et al., 2018).

**TABLE 1:** Description of the five datasets, in which all types of data were transcribed by array, with their fixed *p*-values when annotated to the human databases, and selected matrix-expression-similarity-frequency-based.

Id	Status	Number of samples	Number of genes	P1	P2	P3
E-GEOD-69814	4-Jan-17	11	32,321	10	22	71
E-GEOD-93798	3-Jul-17	42	54,675	11	22	34
GSE108113/E-GEOD-104066	26-Jun-19	76	1,416,100	3	11	53
GSE108113/E-GEOD-108109	17-Jul-18	111	1,416,100	6	23	68
E-GEOD-104948	24-Jan-18	196	76,958	22	51	183
Total genes				52	129	415
Not duplicated				52	129	412
Annotated				33	78	209
Selected				18	45	100

### 3.2 Genetic contributions and their statistical impact on the estimation of predictive models of gene

The results of each number of DE genes were extracted as mentioned in (Figure 1), combined, and estimated in a uniform distribution for the *p* values corresponding to five different datasets (Table 1). Our study was performed based on P3 (*p*-value) for the simple reason that the significance of other selected genes (P2 and P1) is correlated with P3, as visualized in the corresponding figure (Supplementary Figure S1). The results of the ontology analysis are represented as the distance of (dis)/similarity between our list of genes in the interval of (0, 1). When *Sim*(*g*_*i*_, *g*_*j*_) = 1, it means that (*i* = *j*), and when *Sim*(*g*_*i*_, *g*_*j*_)*≅*[0.6, 1], it means that the precision of semantic similarities over genes in *GO*(*g*_*i*_, *g*_*j*_) is more significant and related to common pathologies. Then, when *Sim*(*g*_*i*_, *g*_*j*_)*≅*[0, 0.5]; is referred to the precision of semantic similarities over genes in *GO*(*i*, *j*), which may be significant or related to other common pathologies. However, many genes had a high score on the expression in parallel to a low score of GO (or the reverse). Their classical functional analysis in the literature is based only on the selection of genes from experiments while searching for their dis/similarity score is the input of the classification/regulation analysis in most cases. This problem inspired us to combine/adjust both scores (expression and similarity-based GO annotation) into a single formula that gives us a better chance to predict genes related to our pathologies based on their occurrence frequencies. Consequently, we propose a novel gene selection method by introducing a novel matrix-expression-similarity-frequency-based. Different threshold values give different levels of sensitivity and specificity. Whether the low threshold represented with red color refers to a false positive and the highest with blue color refers to a true positive, as fixed for the validation of our study. This makes it more likely to be specific with more high positives against more sensitive with more low positives, as shown in the corresponding figures (Supplementary Figure S2; Supplementary Tables S1, S2) with a given fixed threshold of observations.

### 3.3 Evaluation and prioritization of tissue using a regression model

An estimating matrix-expression-similarity-frequency-based score for each gene was the input for computing the clustering algorithms (unsupervised learning) to understand biological processes along their molecular functions. Four clusters were found to represent a group of similar observations. To search for shared functions as the first step of supervised learning, all selected genes (in terms of the fixed threshold provided by our prediction analysis) were linked to different databases as enrichment, traits (non-coding variants), tissue databases, and the over-represented conserved transcription factor binding sites based on their score of %GC content. GC content is a measure of the proportion of nucleotides in a DNA sequence that are either guanine (G) or cytosine (C). It is often expressed as a percentage. “GC” is one of the factors considered when identifying over-represented transcription factor binding sites (TFBS) in co-expressed genes Gao et al. (2022). Such analyses generate a mixture of data that requires a biological interpretation. The majority of these genes fall under (kidney, blood, urine, immune system, and embryonic) tissues.

In the hypothesis interpretation and validation section for the GD, we were based on a specific entire to generate a list of the highest information of the gene related to particular biological pathway terms such as the regulation of inflammatory response, regulation of acute inflammatory response, regulation of protein processing/maturation, positive regulation of glomerulus development, of glomerular mesangial cell proliferation, of the adaptive immune response, and complement activation, etc., as shown in the Circos plot (Supplementary Figure S3) as one of the most efficient approaches to visualize genomic data; it allowed us to easily represent all this information on a single plot.

Before evaluating the prioritization model as the second step of supervised learning for our gene clusters, we reanalyzed the highest (more conservative) and lowest threshold (more sensitive) to be sure and to validate the score of a prediction selection, while also justifying that genes selected with the lowest threshold score are not sufficiently expressed in the tissues and/or traits and/or biological processes of interest. A heatmap-like functional classification plot (Supplementary Figure S5) was used to visualize the most significant terms (with the terms expected from the literature), according to some scores, while simultaneously visualizing the sub-ontologies of causative genes as (EGR1, IL33, BMP2, SLAMF8, etc.), in which we filtered/selected the most dominant terms according to our pathology (GD), as well as their GO annotations include (kidney vasculature development, regulation of cell activation, inflammatory, immune effector, adaptive immune, glomerulus, and glomerular mesangial cell proliferation development, etc.).

A bar plot (Supplementary Figure S4) was used to visualize the gene-variant-trait associations among the GWAS catalog used for the most dominant terms such as (complement C3, C4, C7 measurement, and serum IgE/IgA measurement, c-reactive protein measurement, nephrotic syndrome, immune system disease, tuberculosis, glomerular filtration rate, chronic kidney disease, urinary metabolite measurement, C-reactive protein measurement, glomerular filtration rate, etc.). In addition, many genes such as (TNXA, FCER1A, NME3, FMOD, BTG2, PTGER4, AXL, CYP1A2, CYTL1, BHLHE40, IFI16, SPON1, ETNPPL, COL14A1, ITGAV, MYOZ2, CAMK2A, SORT1, RANBP1, etc.) showed some trait association.

Finally, a PieChart plot (Supplementary Figure S6) was used to represent the mean expression of genes in the selected tissues (kidney, renal cancer cell, immune system, urine, blood, blood vessel, blood plasma, hematopoietic stem cell, parenchyma, uroepithelium, HEK 293 EBNA cell, HEK 293ET cell, HK 2 cell).

**FIGURE 5:**
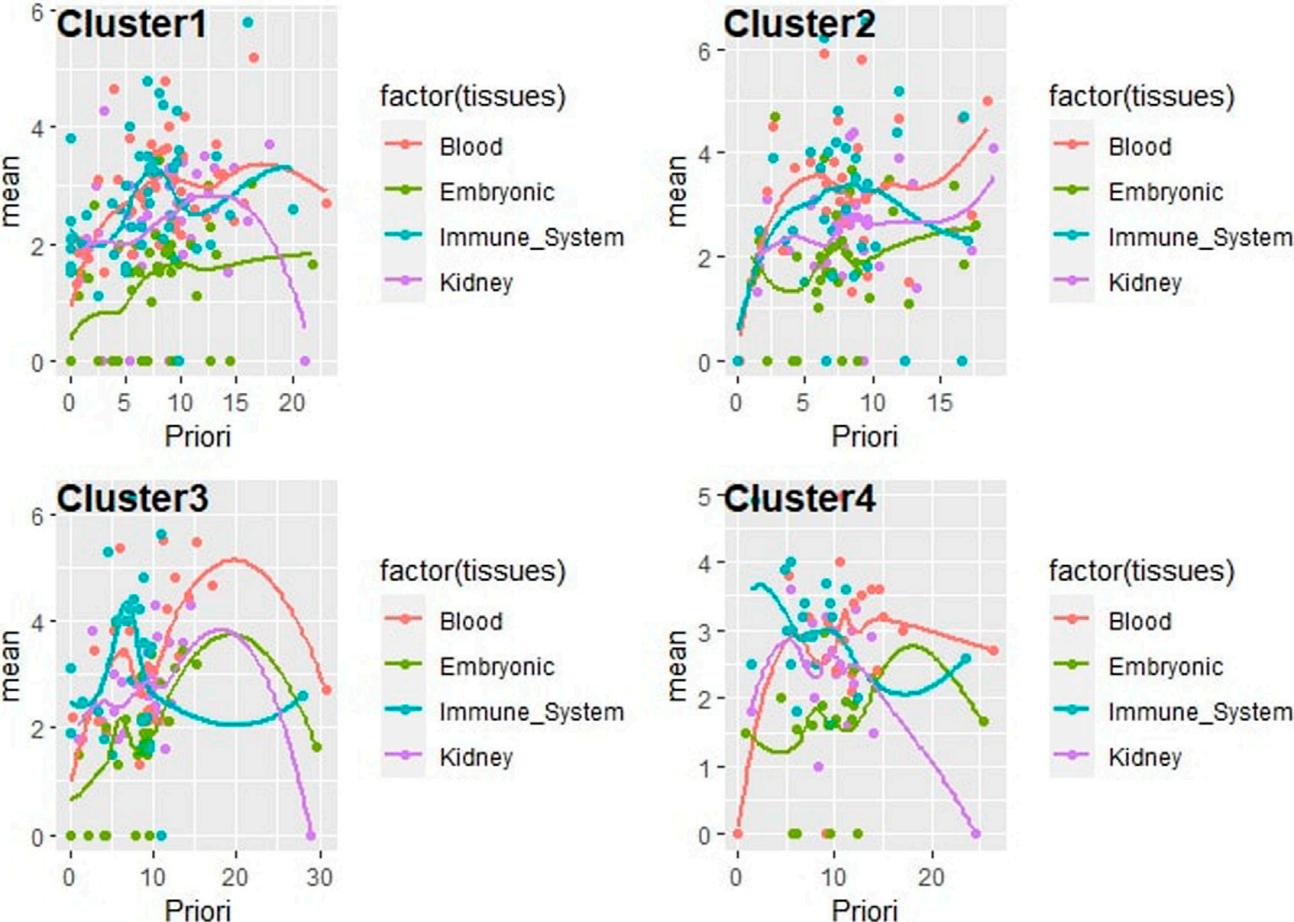
Correlation plot line of the prioritization model as logistic regression for gene clusters consisting of a new scoring including expression, similarity, and frequency from mean expression of various tissues of interest, under each element of the set (phenotype/trait, and enhancer/promoter).

The gene prioritization model could be formulated as follows (two parts): arrange candidate genes in the order of their normalized scores and frequencies of occurrence from matrix-expression-similarity-frequency-based, then to their normalized scores and frequencies of occurrence for categorical tissues, biological processes, TFs, and variants. The logistic regression model (parametric regression) was performed on categorical data to prioritize the dependent variable using a given set of independent variables to solve classification problems. In logistic regression, linear connections between the dependent and independent variables are not needed. However, there should be no collinearity between the independent variables. As discussed above, logistic regression was used to classify the elements of each cluster under different tissues by calculating the mean of the normalized expression of each set. A confidence regression line provides a representation of the uncertainty in the sense that, among a cluster of genes, we can prioritize the most promising ones based on their prioritization scores on each selected tissue, as visualized in (Figure 5). Many redundant genes were more promising by applying the priority model to fixed tissues, as noted in the table (Table 2). This has only one interpretation, which has great value in the expression in the different tissues analyzed. The process of arranging all possible prioritizing disease-causing genes based on their logistic regression has shown that consistent genes may reside in distinct pathways and affect the promoter/regulatory region of location-related organisms. Based on PubMed resources, 972 abstracts were extracted and approved that included 11 genes (COL4A5, EGR1, GDF15, CPE, CASK, NT5E, JUN, AXL, CCL3, IL33, ITGAV) from our candidate genes that have already been reported in previous studies on GD.

**TABLE 2:** The most promising genetic head data prioritization-models for the four clusters.

Cluster N 1				
Kidney	Urine	Immune system	Blood	Embryonic dev
IL33	SPON1	SPON1	SPON1	SPON1
RANBP1	IL33	IL33	IL33	IL33
ANK1	RANBP1	RANBP1	RANBP1	RANBP1
MYOZ2	ANK1	ANK1	MYOZ2	ANK1
MEOX1	MYOZ2	MYOZ2	PALB2	PALB2
CACNA1G	MEOX1	MEOX1	CACNA1G	MYOZ2
PALB2	CACNA1G	CACNA1G	ANK1	MEOX1
Cluster N 2				
Kidney	Urine	Immune system	Blood	Embryonic dev
BMP2	BMP2	COL15A1	BMP2	BMP2
COL15A1	COL15A1	ITGAV	COL15A1	COL15A1
ITGAV	ITGAV	BMP2	ITGAV	ITGAV
ATP13A3	ATP13A3	ATP13A3	ATP13A3	ATP13A3
TNFSF10	TNFSF10	TNFSF10	TNFSF10	TNFSF10
FGF7	FGF7	FGF7	FGF7	FGF7
Cluster N 3				
Kidney	Urine	Immune system	Blood	Embryonic dev
SPON1	SPON1	SPON1	SPON1	SPON1
RET	RET	RET	RET	RET
FOS	FOS	FOS	FOS	FOS
SFRP4	SFRP4	SFRP4	AXL	AXL
AXL	NME3	P2RY14	SFRP4	SFRP4
NME3	CA10	NME3	GDF15	GDF15
CA10	BTG2	CA10	NME3	CCL4
Cluster N 4				
Kidney	Urine	Immune system	Blood	Embryonic dev
SPON1	SPON1	SPON1	SPON1	SPON1
MYOZ2	MYOZ2	MYOZ2	TDG	TDG
PALB2	PALB2	PALB2	PALB2	PALB2
SORT1	RAP1B	RAP1B	SPRY1	MYOZ2
RAP1B	NME3	P2RY14	MYOZ2	SORT1
TDG	SORT1	NME3	RAP1B	SPRY1

### 3.4 Genetic interactions bridging transcription factors and pathways in genome-wide association studies

Traditional statistical methods consider gene-gene interactions and estimate interactions among only a fixed or small number of phenotypes information with significant main effects. However, our MGTI algorithm-based model can be applied when the data are highly dimensional (many attributes or independent variables) or when interactions between more than two tissues may play a role in human disease etiology and regulation data mining analysis. To perform ETL (extract-transform-load) operations, a vector of SQL commands was used to select data based on a specific entity, focusing on choosing the best score of gene-gene interaction and supporting and validating the hypothesis validation. Representatives Table 3 show some random gene-gene interaction algorithm tools based MGTI model.

**TABLE 3:** Global summary of the mixed-gene tissue interaction (MGTI) model based on gene prioritization results and randomly selected genes from the results.

Cluster N 1		
	IL 33: Interleukine 33	Genes 2: RANBP1: RAN binding protein 1
Mean-coExp (adjusted)	0.17	0.87
Mean-tissues	5.8	2.4
Description-tissues	“Immune system”	“Kidney”
Mean-BP	0.59	-
Description-BP	“Regulation of: inflammatory response.” of immune effector process.“response to external stimulus.” cytokine production. “inflammatory response.” adaptive immune response based on somatic recombination of immune receptors built from immunoglobulin superfamily domains.” proteolysis.” adaptive immune response.” neuroinflammatory response.” of cell activation.”	-
Mean-TFs	0.77	1.54
Description-TFs	“Myc”, “CEBPA”, “USF1”, “TBP”	“Zfx”, “MYC::MAX”
		“Mycn”, “RXRA::VDR”
	“FOXO3”, “Arnt::Ahr”, “Sox5”	“ZNF354C″, “Myc”, “Egr1”
Mean-GWAS	4.75	0.4
Description-GWAS	“Acute kidney injury”	“Urinary metabolite
		Measurement”
		“Chronic kidney disease”
		“Blood protein measurement”
		“Platelet count”
		“Serum IgG glycosylation
		Measurement”
Prioritization score	16.53	16
Interaction score: 0.75		
Cluster N 2		
	Genes 1: ITGAV: Integrin alpha V	Genes 2: FCER1A: Fc fragment of IgE
Mean-coExp	1.05	1.69
Mean-tissues	3.35	1.9
Description-tissues	“Hematopoietic stem cell” “Parenchyma”	“Kidney”
Mean-BP	0	0
Description-BP		
Mean-TFs	1.18	0.96
Description-TFs	“IRF2”, “Sox17”, “Esrrb”, “ARID3A″	“NFYA”, “NHLH1”, “Gata1”
	“RORA1”, “SOX9”	“Myf”, “FEV”
Mean-GWAS	2.38	0.003
Description-GWAS	“Urinary albumin to creatinine ratio”	“C-reactive protein measurement”
	“Microalbuminuria”	“Leukocyte count”
		“Serum IgE measurement”
Prioritization score	16.01	6.6
Interaction score: 0.63		
Cluster N 3		
	Genes 1: PHLDA2: Pleckstrin Homology Like Domain Family A Member 2	Genes 2: BTG2: BTG Anti-Proliferation Factor 2
Mean-coExp	0.96	0.23
Mean-tissues	2.4	3.15
Description-tissues	“Kidney”	“Blood”
		“Blood vessel”
Mean-BP	-	-
Description-BP	“Regulation of binding”	-
	“Regulation of protein binding”	
Mean-TFs	1.19	1
Description-TFs	“GABPA”, “NFE2L2”	“INSM1”, “NFYA”, “Tcfcp2l1”
	“IRF1”, “ELK4”	“CREB1”, “Zfx”, “Klf4”
	“Egr1”, “FEV”	
	“RELA”	
Mean-GWAS	-	0.49
Description-GWAS	-	“Mean corpuscular hemoglobin”
		“Red blood cell distribution width”
		“Immunoglobulin isotype switching
		Measurement”
		“Multiple sclerosis”
Prioritization score	10.17	9.26
Interaction score: 0.65		
Cluster N 4		
	Genes 1 : MYOZ2 : Myozenin 2	Genes 2: SORT1: Sortilin 1
Mean-coExp	1.8	3.15
Mean-tissues	1.5	1.7
Description-tissues	“Kidney”	“Urine”
Mean-BP	-	-
Description-BP	-	-
Mean-TFs	1.08	1.5
Description-TFs	“PLAG1”, “MEF2A″	“RORA2”, “MEF2A″, “RREB1”
	“ELF5”, “Myb”, “FOXD1”	“NFYA”, “CREB1”, “Gfi”
Mean-GWAS	2.27	0.06
Description-GWAS	“Body height” “glomerular filtration rate” “serum IgE measurement”“renal transplant outcome measurement” “donor genotype effect measurement”	“Blood protein measurement” “C-reactive protein measurement” “glomerular filtration rate” “C-reactive protein measurement” “creatinine measurement” “body height” “chronic kidney disease”
Prioritization score	13.9	11.7
Interaction score:0.7		

Based on the interaction between IL33 and RANBP1 genes, which are expressed in the immune system and kidney tissue, respectively, they contribute to indirect immune-mediated renal disease, often many acute forms of renal disease, and play a central role in the progression of chronic kidney disease. When the interaction between these genes is high, the network interaction leads to new diagnostic methods and treatment solutions to inhibit further progression and promote appropriate tissue repair.

A glomerular filtration rate, expressed between urine and kidney tissue, checks how well the kidneys are working. The kidneys are two organs on either side of the spine, near the waist. They have tiny filters called glomeruli. These filters remove waste and extra water from the blood and get rid of them through urine. When the kidneys are damaged by kidney disease, they cannot filter blood as fast as they should. The interaction between the MYOZ2 and SORT1 genes can be used to check for kidney disease by measuring how much blood is filtered in the kidneys and how much C-reactive protein is increased.

Finally, the gene interaction results encode a superfamily of proteins that plays a role in transcriptional expression, whether ligands of this family bind various enzyme binding and receptors/initiators leading to recruitment and activation of family transcription and signaling factors regulating the level and stability of gene expression. The encoded proteins possess different motifs composed of intracellular and extracellular domains. Several cellular functions may be involved in multiple cell types and various tissues. While alternative splicing results in multiple transcript variants of these candidate genes. While alternative splicing results in multiple transcript variants of these candidate genes.

Based on a suite of queries such as network analysis, functional enrichment analysis, and cross-validation with network analysis that represent different types of analyses performed on the data warehouse comparing the results to previously validated ones. The currently proposed search validation approach to gene prioritization and data warehouse results is based on selecting these improved best interaction scores to solve the sequencing analysis. The best-improved scores were considered biomarker modules to detect and rank novel forms of the activation of glomerular disease genes.

The early diagnosis and prognosis of any type of disease are correlated with the need for bioinformatics tools, as they can facilitate the subsequent clinical management of patients, in which the follow-up analysis can be applied to other types of data such as cancer data, etc. Finally, our global objective was to incorporate/develop a Shiny web-based application as an R framework designed to help and facilitate users’ navigation under “Shiny apps” as to test our algorithms tools: gene prediction, prioritization, and interaction.

## 4 Conclusion

The long-term goal of this research is to improve our understanding of the molecular/biological mechanisms of activation and regulation of a set of novel/common genes implicated in our pathology in different target cells. To address the above issues, we proposed three contributions combining and adjusting multiple similarity scores of gene expression gene ontology terms based on similarity scores, which were explained by our algorithm as essential prediction tasks for evaluating the regulatory pathways. Then, machine learning techniques to prioritize candidate genes were demonstrated. Finally, some significant genetic interactions were detected as a validation of the results by applying our algorithm model.

The linked resources of biological/clinical data-based expression profiles (adjusted scores) are used to validate molecular biology research. Experimental validation of all associations facilitates the discovery of causative genes related to glomerular diseases (GD). Genes such as EGR1, IL33, BMP2, and SLAMF8 have their GO annotations such as kidney vasculature development, regulation of cell activation/inflammation/immune effectors/adaptive immune/glomerulus/glomerular mesangial cell proliferation] development, etc.

Other genes such as TNXA, FCER1A, NME3, FMOD, BTG2, PTGER4, AXL, CYP1A2, CYTL1, BHLHE40, IFI16, SPON1, ETNPPL, COL14A1, ITGAV, MYOZ2, CAMK2A, SORT1, RANBP1, in which their variants information include complement a set of C(3,4,7) protein measurement, serum IgE/IgA measurements, c-reactive protein measurement, nephrotic syndrome, immune system disease, tuberculosis, glomerular filtration rate, chronic kidney disease, etc.

The latter enables a rapid interpretation of complex gene expression studies and illustrates an overview of a computational model for gene prioritization and their genetic interactions.

Finally, the majority of our prioritized genes fall under transcription co-factor binding, regulation of glomerular mesangial cell proliferation, regulation of adaptive immune response, complement activation, etc.

As a future area of study, new deep neural network algorithms are proposed to summarize the clustering of genes based on their regulatory pathway results, not only on their expression. This can be challenging due to the many gene ontology (GO) terms connected as directed acyclic graphs. This new area of analysis can bring about changes in molecular, cellular, and biological processes. Finally, we demonstrated that genotype-phenotype associations can be adjusted and updated by using our feed and back-propagation algorithms, which minimize the loss function for gene ontology (GO) terms.

## Data Availability

The datasets and R Shiny application presented in this study can be found in online repositories: https://github.com/boutaina-ettetuani/Glomerular-Diseases-Analysis Accession: (GSE69814, GSE93798, GSE108113, GSE104948).
