# Supplementary material for: Adjustment of p-value expression to ontology using machine learning for genetic prediction, prioritization, interaction, and its validation in glomerular disease
Source: Front Genet. 2023 Oct 12;14:1215232. doi: 10.3389/fgene.2023.1215232 (PMC10603191; doi:10.3389/fgene.2023.1215232)
Supplement: Supplementary file 1 [file DataSheet1.PDF]

Supplementary Material

LIST OF ABBREVIATIONS

- Gene Ontology (GO)
- Glomerular Diseases (GD)
- C3 Glomerulopathy (C3G)
- Differential Expression (DE)
- NCBI Gene Expression Omnibus (GEO)
- Robust Multi-array Average (RMA)
- Information Content (IC)
- Directed Acyclic Graph (DAG)
- Best-Match Average (BMA)
- Genome-Wide Association Study (GWAS)
- Transcription Factors (TFs)
- Extracting Transforming, and Loading (ETL)
- A Primary Foreign Key (PFK)
- A Primary Key (PK)
- Foreign Key (FK)
- Mixed-Gene Tissues Interaction (MGTI)

1 SUPPLEMENTARY TABLES AND FIGURES

1.1 Figures

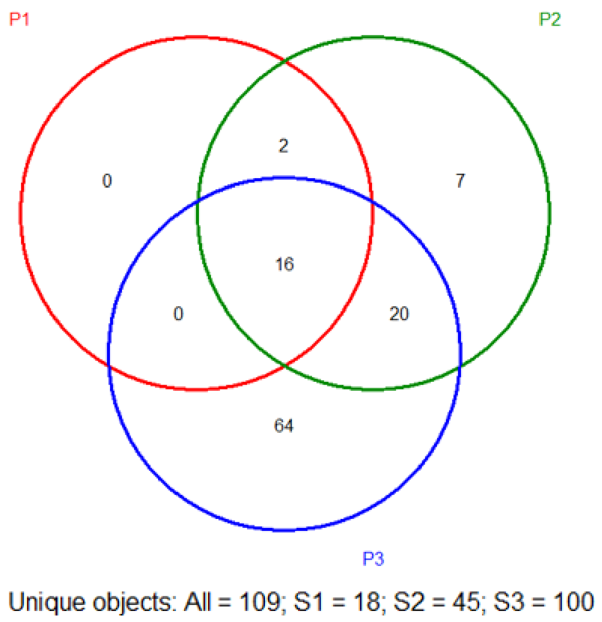

Figure S1. Venn diagram showing the correlation of DE genes result between the five datasets used in this study showing 16 causative genes known to be related to glomerular diseases (GD).

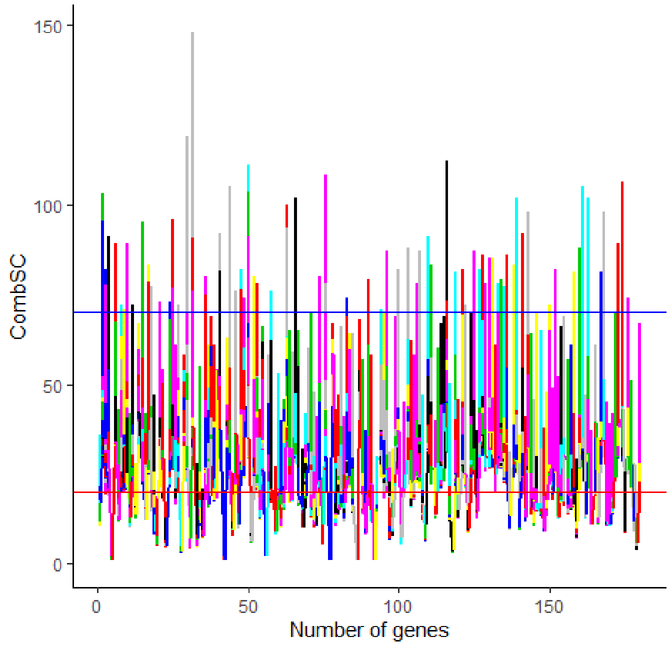

Figure S2. Visualization methods: Adjusted expression to similarity scores (based on P3), and their frequency positions with a fixed low vs. high threshold strategy used to validate the matrix-Expression-Similarity-Frequency-based.

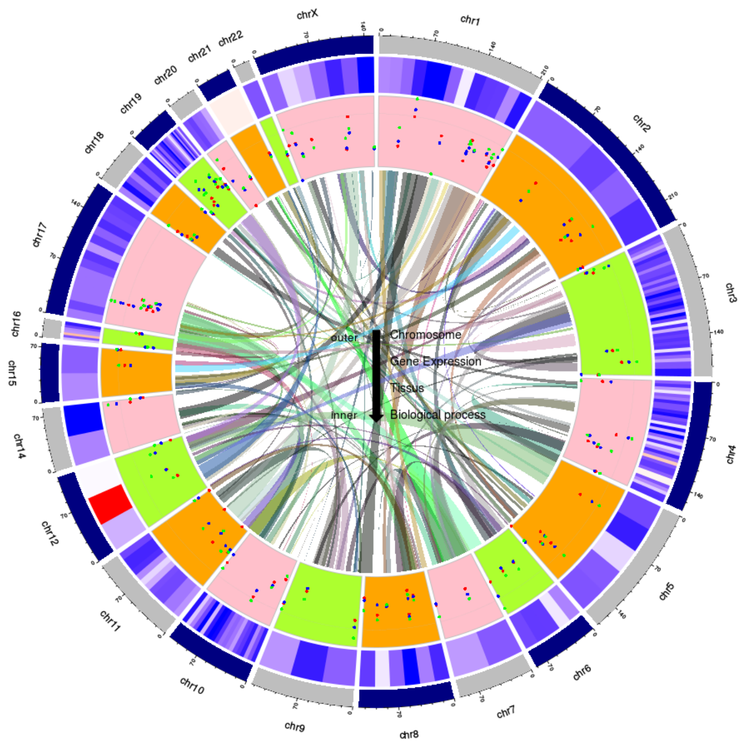

Figure S3. Circos plot created with shinyCircos. Chromosome ideogram representation from candidate genes as the first track, gene expression results according to their P-values as the second track, tissues results according to their normalized expression scores (kidney, blood, urine, immune system, and embryonic) as the third track, and finally all biological process relation that links our candidate's genes as the fourth track.

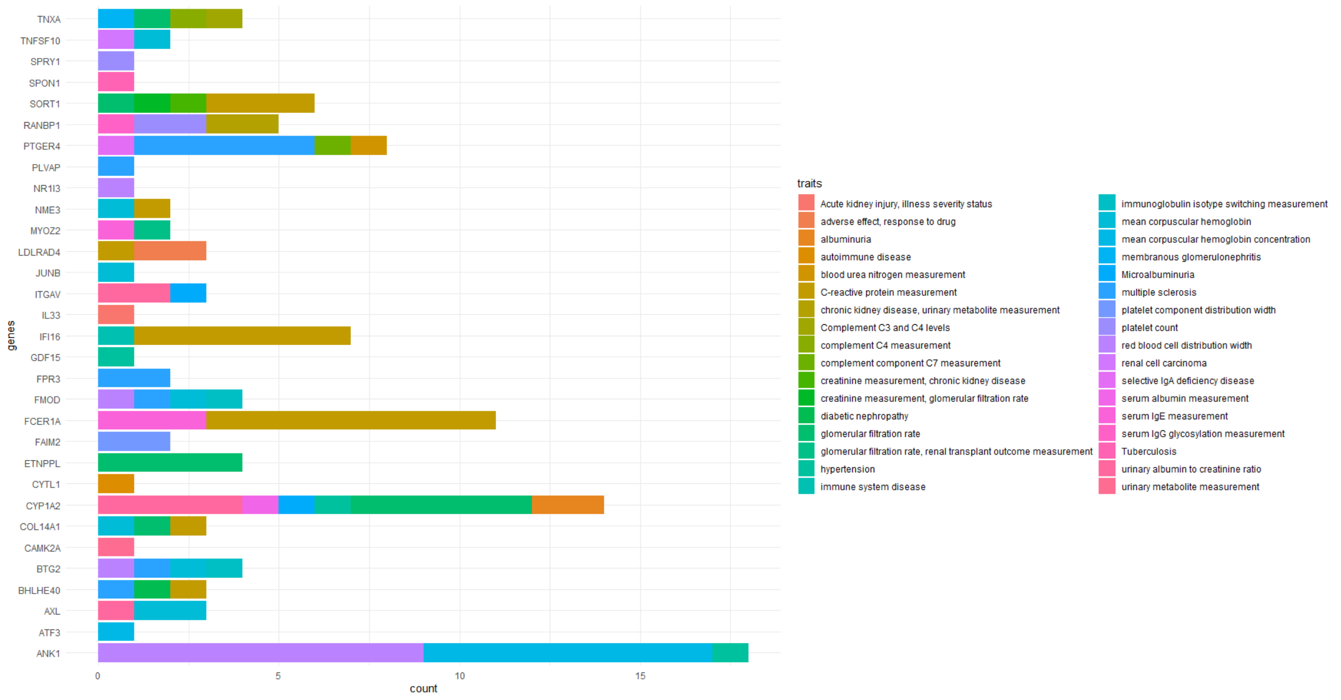

Figure S4. Highest threshold visualization: GWAS Catalog presentation was used with the retrieval of important traits whose color of the bar plot indicates the occurrence frequency of each entity.

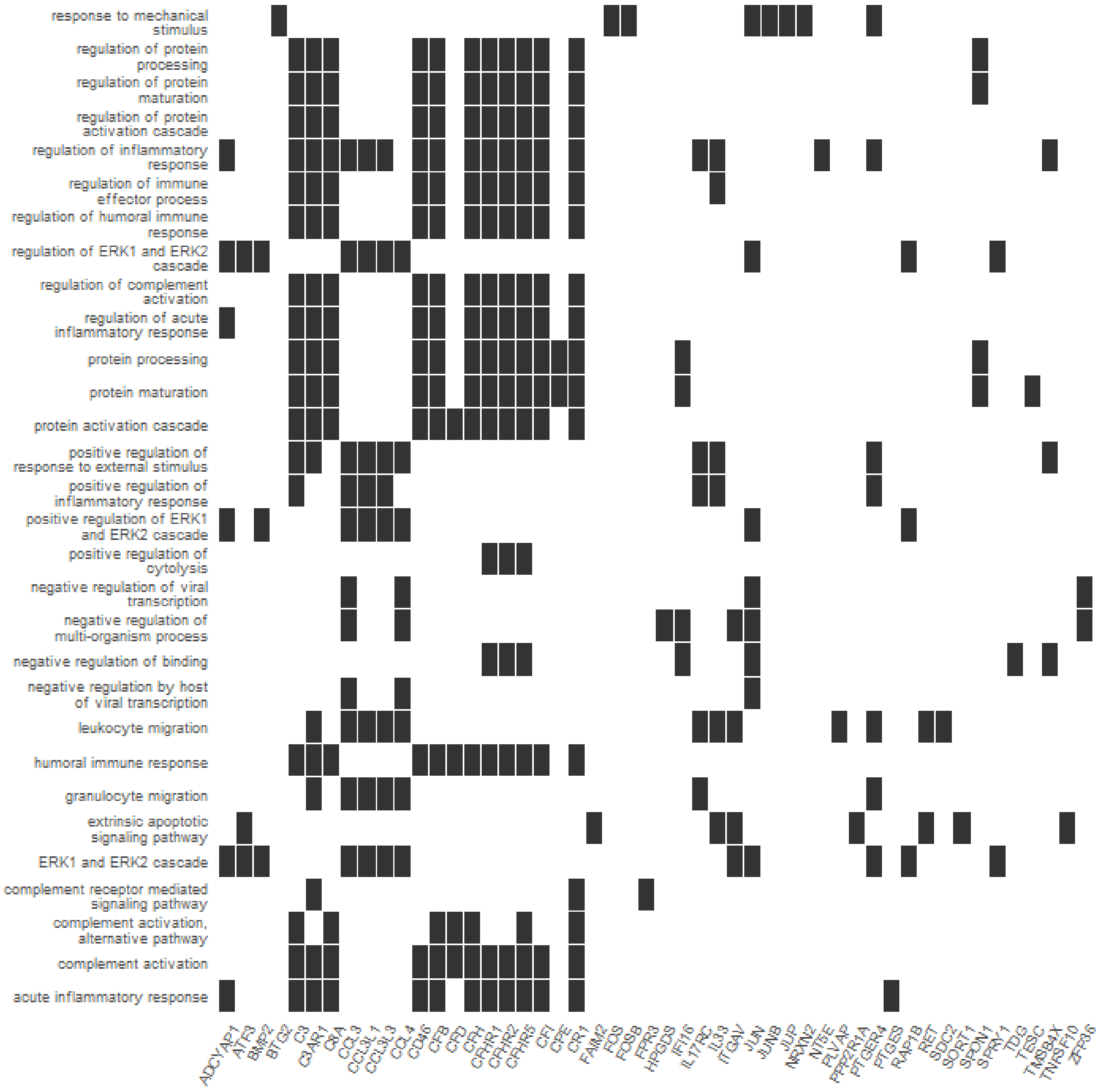

Figure S5. Highest threshold visualization: The top pathways displayed for all enriched terms with specifics scores visualizing the linkage of genes and biological concepts as a network for the literature genes (C3, ADAM19, ADAMTS13, C3AR1, C8A, CD46, CFB, CFD, CFI, CFHR (1-5), CFH, CD46), and candidates genes.

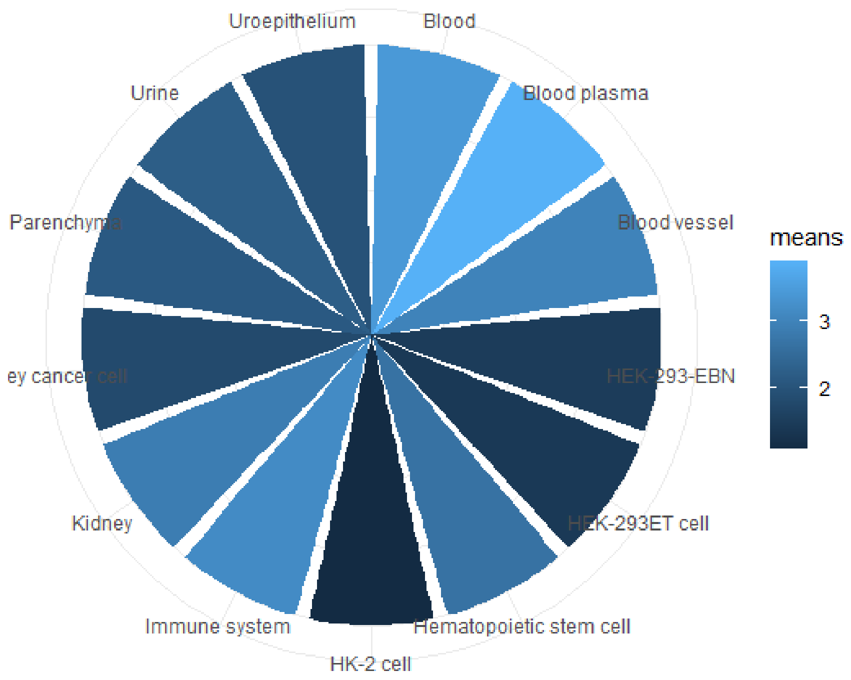

Figure S6. Highest threshold visualization: A Pie Chart plot is used to represent the means of expression of genes in the selected tissues related to glomerular disease.

Table S1. Description of the top DE genes selected based by their frequency, normalized value of expression score from experiences and normalized value of similarity score forming a final score of selection

”SYMBOL”	”EntrezID”	”Similarityscore”	”Expressionscore”	”FreqRow”	”Selectionscore”
”BTG2”	7832	0.201404109	0.491	148	0.23417416
”NT5E”	4907	0.201404109	0.514	148	0.532948089
”PALB2”	79728	0.201404109	0.829	148	4.624851895
”RBM39”	9584	0.201404109	0.635	148	2.104758757
”TDG”	6996	0.201404109	1	148	6.846171104
”SPRY1”	10252	0.19295318	1	119	4.300799796
”SARAF”	51669	0.277777051	0.654	112	0.285081586
”SIK1”	150094	0.277777051	1	112	3.686399825
”CALML3”	810	0.256227506	0.875	111	2.380799887
”CYTL1”	54360	0.256227506	1	111	3.598628401
”HPGDS”	27306	0.256227506	1	111	3.598628401
”PTGES”	9536	0.256227506	0.82	111	1.844955341
”TESC”	54997	0.238212196	1	108	3.335314128
”P2RY14”	9934	0.268035003	1	106	3.159771279
”SORT1”	6272	0.268035003	1	106	3.159771279
”FOSB”	2354	0.253073316	1	105	3.071999854
”MYOZ2”	51778	0.253073316	1	105	3.071999854
”STMN2”	11075	0.217321929	1	105	3.071999854
”TNXA”	7146	0.253073316	1	105	3.071999854
”CPE”	1363	0.284246248	1	103	2.896457006
”CA10”	56934	0.271693807	0.769	102	0.740615279
”IL17RC”	84818	0.243914901	0.782	102	0.857000188
”PLVAP”	83483	0.273926882	1	102	2.808685581
”SPOCK1”	6695	0.271693807	1	102	2.808685581
”SPON1”	10418	0.271693807	1	102	2.808685581
”TMSB4X”	7114	0.243914901	1	102	2.808685581
”RET”	5979	0.337914199	1	100	2.633142732
”TPSB2”	64499	0.337914199	0.89	100	1.667657064
”AXL”	558	0.293897613	1	98	2.457599884
”NME3”	4832	0.257548252	1	98	2.457599884
”RAP1B”	5908	0.270444831	1	96	2.282057035
”CASK”	8573	0.229363578	1	95	2.19428561
”ETNPPL”	64850	0.250840883	0.863	92	0.824700304
”PIP4K2A”	5305	0.250840883	1	92	1.930971337
”SFRP4”	6424	0.311987601	1	92	1.930971337
”CACNA1G”	8913	0.204894184	1	91	1.843199913
”FJX1”	24147	0.323855695	1	91	1.843199913
”GDF15”	9518	0.323855695	1	91	1.843199913
”CYP1A2”	1544	0.227755641	1	89	1.667657064
”FMO3”	2328	0.227755641	1	89	1.667657064
”NR1I3”	9970	0.367062067	1	89	1.667657064
”PHLDA2”	7262	0.32651486	1	89	1.667657064
”PPP2R1A”	5518	0.32651486	1	89	1.667657064
”DST”	667	0.295416923	1	88	1.579885639
”FCER1A”	2205	0.327276311	1	88	1.579885639
”FOS”	2353	0.327276311	1	88	1.579885639
”FAIM2”	23017	0.228267191	1	87	1.492114215
”LDLRAD4”	753	0.23888445	1	87	1.492114215
”TCIM”	56892	0.281570667	1	87	1.492114215
”ATP8B1”	5205	0.257349884	1	86	1.404342791

Table S2. Table S1 continued

"SYMBOL"	"EntrezID"	"Similarityscore"	"Expressionscore"	"FreqRow"	"Selectionscore"
"CCL4"	6351	0.345380863	1	86	1.404342791
"CCL3L1"	6349	0.308248619	1	85	1.316571366
"CCL3L3"	414062	0.308248619	1	85	1.316571366
"ATF3"	467	0.262923344	1	83	1.141028517
"IL3RA"	3563	0.286988304	1	83	1.141028517
"COL14A1"	7373	0.308962271	1	82	1.053257093
"COL15A1"	1306	0.308962271	1	82	1.053257093
"COL4A5"	1287	0.308962271	1	82	1.053257093
"COL8A2"	1296	0.308962271	1	82	1.053257093
"FGF7"	2252	0.316509131	1	82	1.053257093
"ITGAV"	3685	0.308962271	1	82	1.053257093
"JUNB"	3726	0.309405244	1	82	1.053257093
"KRT17"	3872	0.315713233	1	82	1.053257093
"NR4A2"	4929	0.358466375	1	82	1.053257093
"RBM7"	10179	0.233947974	0.948	82	0.678999739
"TIA1"	7072	0.233947974	1	82	1.053257093
"BHLHE40"	8553	0.266890509	1	81	0.965485669
"CAMK2A"	815	0.335603013	1	81	0.965485669
"PON2"	5445	0.194113088	1	81	0.965485669
"ADGRG2"	10149	0.251778894	1	80	0.877714244
"DLGAP1"	9229	0.302858711	1	80	0.877714244
"GABRE"	2564	0.302858711	0.907	80	0.224694846
"GABRP"	2568	0.302858711	1	80	0.877714244
"RANBP1"	5902	0.265220164	1	80	0.877714244
"BMP2"	650	0.326475658	1	79	0.78994282
"PLPP2"	8612	0.203451096	1	79	0.78994282
"ADCYAP1"	116	0.335493919	1	78	0.702171395
"ATP13A3"	79572	0.325452888	1	78	0.702171395
"CCL3"	6348	0.325452888	1	78	0.702171395
"EGR1"	1958	0.285667103	1	78	0.702171395
"MEOX1"	4222	0.249930431	1	78	0.702171395
"FLRT3"	23767	0.29124273	1	77	0.614399971
"SCLY"	51540	0.189923511	1	77	0.614399971
"FMOD"	2331	0.218560324	1	76	0.526628546
"SDC2"	6383	0.282266952	1	76	0.526628546
"CHRNA1"	1134	0.309599595	1	74	0.351085698
"JUP"	3728	0.311112338	1	74	0.351085698
"TNFSF10"	8743	0.335372076	1	74	0.351085698
"NRXN2"	9379	0.301758332	1	73	0.263314273
"IFI16"	3428	0.315685463	1	72	0.175542849
"IL33"	90865	0.272561704	1	72	0.175542849
"PTGER4"	5734	0.336745052	1	72	0.175542849
"ZFP36"	7538	0.28617859	1	72	0.175542849
"ADRA2A"	150	0.330245158	1	71	0.087771424
"RGS2"	5997	0.274444422	1	71	0.087771424
"ANK1"	286	0.205208436	1	70	0.01
"DIO2"	1734	0.181527103	1	70	0.01
"FPR3"	2359	0.339467003	1	70	0.01
"JUN"	3725	0.337166312	1	70	0.01
"SRPX2"	27286	0.259576913	1	70	0.01
